# Determinants of virological failure among patients on first line highly active antiretroviral therapy (HAART) in Southwest Ethiopia: A case-control study

**DOI:** 10.3389/fpubh.2022.916454

**Published:** 2022-10-25

**Authors:** Biruk Bogale, Adane Asefa, Alemnew Destaw, Gachana Midaksa, Zufan Asaye, Mathewos Alemu Gebremichael, Asrat Arja Wolde, Ejig Yimer, Tewodros Yosef

**Affiliations:** ^1^School of Public Health, College of Medicine and Health Sciences, Mizan-Tepi University, Mizan-Aman, Ethiopia; ^2^Department of Statistics, College of Natural Sciences, Mizan-Tepi University, Tepi, Ethiopia; ^3^School of Public Health, College of Medicine and Health Sciences, Arba Minch University, Arba Minch, Ethiopia; ^4^Department of Data Repository and Governance, National Data Management Center for Health, Ethiopian Public Health Institute, Addis Ababa, Ethiopia; ^5^Department of Public Health, Mizan-Aman Health Science College, Mizan-Aman, Ethiopia

**Keywords:** HIV/AIDS, virological failure, HAART, case-control, Ethiopia

## Abstract

**Background:**

Virological failure remains a public health concern among patients with human immunodeficiency virus (HIV) after treatment initiation. Ethiopia is one of the countries that aims to achieve the global target of 90-90-90 that aims to achieve 90% virological suppression, but there is a paucity of evidence on the determinants of virological failure. Therefore, the study is intended to assess determinants of virological treatment failure among patients on first-line highly active antiretroviral therapy (HAART) at Mizan Tepi University Teaching Hospital (MTUTH), Southwest Ethiopia.

**Method:**

A hospital-based unmatched case-control study was conducted from 11 November to 23 December 2020, among 146 cases and 146 controls. All cases and controls were selected randomly using computer-generated random numbers based on their medical record numbers. During the document review, data were collected using checklists, entered into Epi-data version 4.0.2, and analyzed by SPSS version 25. A multivariable logistic regression analysis was done to identify the independent determinants of virological treatment failure.

**Results:**

In this study, being male (adjusted odds ratio (AOR) = 1.89, 95% CI: 1.04, 3.47), substance use (AOR = 2.67, 95% CI: 1.40, 4.95), baseline hemoglobin (Hgb) < 12 mg/dl (AOR = 3.22, 95% CI: 1.82, 5.99), poor drug adherence (AOR = 3.84, 95% CI: 1.77, 5.95), restart ART medication (AOR = 2.45, 95% CI: 1.69, 7.35), and opportunistic infection (OI) while on HAART (AOR = 4.73, 95% CI: 1.76, 12.11) were determinants of virological treatment failure.

**Conclusion:**

The study revealed that the sex of the patient, history of substance use, baseline Hgb < 12 mg/dl, poor drug adherence, restart after an interruption, and having OI through the follow-up period were determinants of virological failure. Therefore, program implementation should consider gender disparity while men are more prone to virological failure. It is also imperative to implement targeted interventions to improve drug adherence and interruption problems in follow-up care. Moreover, patients with opportunistic infections and restart HAART need special care and attention.

## Background

The human immunodeficiency virus (HIV) continues to be a global public health challenge, affecting over 38 million people and infecting 1.5 million new people at the end of 2020 (1). Despite the decreased incidence of HIV infection in Ethiopia, more than 616,105 people are estimated to live with the virus in 2020, which puts the country among the worst affected in sub-Saharan Africa (2).

Mortality due to HIV has decreased since the era of highly active antiretroviral therapy (HAART). Evidence has shown that individuals on HAART with an undetectable viral load, the absence of an advanced clinical finding, and a high CD4 count are less likely to transmit HIV to other people and reduce HIV-associated morbidity and mortality. However, due to many factors, patients experience deterioration and treatment failure (3).

Virological failure is defined as a persistently detectable viral load exceeding 1,000 copies/ml (i.e., two consecutive viral load measurements within a 3-month interval, with adherence support between measurements) after at least 6 months of starting a new ART regimen (4). Patients who experienced virological failure were found to have an increased risk of clinical progression to AIDS and mortality when compared with patients with a complete virological response (3). Virological failure is a more informative parameter to determine treatment failure and is a common problem among patients on first-line ART (3, 5).

Optimization of viral load testing capacities is an important measure for the global control of the HIV epidemic, particularly in resource-limited countries, such as Ethiopia (6). Studies showed that virological failure is common in different countries, particularly in Sub-Saharan countries: 32% in Malawi (7), 16% in Swaziland (8), 24.6% in Kenya (9), 24% in Mozambique (10), 41.3% in Gabon (11), 11.9% in Rwanda (12), 11% in Uganda (13), and 14.8% in Ethiopia (14).

Globally, the United Nations Program on HIV/AIDS (UNAIDS) 90-90-90 is committed to having 90% of people on HAART virally suppressed by 2030, and as a result, HIV treatment failure would be prevented. Despite this ambitious goal, according to a systematic analysis of national HIV treatment cascades in 69 countries by 2016, viral suppression was between 7% in China and 68% in Switzerland (15). In Sub-Saharan countries, 83% were virally suppressed, contributing to a 29% reduction in new HIV infections between 2010 and 2016 (16). Mathematical modeling predicts that if the levels of NNRTI drug resistance exceed 10% in sub-Saharan Africa, drug resistance is predicted to be responsible for an additional 105,000 new HIV infections, 135,000 AIDS deaths, and US$ 650 million in ART drug costs between 2016 and 2020 (16).

Ethiopia has limited resources available for diagnosing treatment failure and monitoring patient response with viral load, which is the gold standard method for monitoring treatment effectiveness. Nevertheless, this is not feasible since patients initiate treatment with very advanced diseases. Therefore, identifying the determinants of virological failure and reducing its incidence would help to realize the 90-90-90 treatment target and achieve sustainable development goal 3. It is also important for the patients to prevent the progression of AIDS and have better efficacy of the second-line regimen. However, there is an evidence gap in identifying the determinants in the study area.

Therefore, this study aimed to assess determinants of virological treatment failure among patients on first-line HAART at Mizan Tepi University Teaching Hospital (MTUTH) and provide a wider understanding of the determinants of virological treatment failure, and enable early identification and control of this factor, which results in decreasing patients' risk of treatment failure.

## Method and material

### Study design and setting

A hospital-based case-control study was conducted from November to December 2020 at the Mizan Tepi University Teaching Hospital ART clinic. The hospital is located in Mizan-Aman town, which is located 561 km away from Addis Ababa toward the southwest of the country. The hospital has been serving more than 3 million people from two regions in southwestern Ethiopia. There were 3,805 registered patients on HAART from 2012 to 2020 in the ART clinic. The clinic provides voluntary counseling and testing (VCT), provider imitative counseling and testing (PICT), and prevention of mother to child transmission (PMTCT) services.

### Study population

All adult patients infected with HIV (>15 years) who had taken ART for at least 6 months with two consecutive documented viral load test results at MTUTH between December 2012 and November 2020 were our source populations. Patients who had viral load results >1,000 or more in two consecutive viral load tests were considered as cases, and controls were those who had viral load results <1,000 in two consecutive viral load tests.

### Sample size and sampling procedure

The sample size was determined using Epi Info 7.2 Stat-Cal by taking age as a predictor variable for virological failure based on the study done in Gonder University Referral Hospital (3) assuming a 1:1 case to control ratio, 5% margin of error, and 80% of study power. The final calculated sample size was 292 (146 cases and 146 controls). After filtering the illegible patients' cards, sampling frames were generated using ART medical record numbers for both cases and controls separately. There were a total of 207 patients with viral load results >1,000 or more and 3,521 patients who had viral load results <1,000 in the study period. Finally, cases and controls that fulfilled the inclusion criteria were randomly selected using computer-generated random numbers based on the medical record number of the patient. Cases and controls with 20% missing study variables were excluded from the study.

### Study variables

The dependent variable (outcome of interest) was a virological failure. Age, marital status, sex, religion, educational status, place of residence, substance use, adherence, non-disclosure of HIV status, baseline WHO staging, baseline CD4 count, chronic illness, baseline hemoglobin (Hgb), history of opportunistic infection (OI), baseline body mass index (BMI), duration on ART, ART regime, previous exposure to ART, and restart after interruption were independent variables.

### Data collection method

Data were extracted *via* document review using a structured checklist prepared in English adapted from the Ethiopian Federal Ministry of Health ART clinic intake and follow-up form, and were collected by the three health officers after consent was received from the MTUTH medical director office, the ART department, and card room to extract cards and to get the necessary information. The information was extracted from the patient's cards.

### Data quality control

To assure the quality of data, we used the Federal Ministry of Health (FMOH) ART clinic intake checklist, and the collected data were reviewed and checked for completeness before data entry.

### Operational definition

**Virological failure:** Viral load above 1,000 copies/ml based on two consecutive viral load measurements in 3 months, with adherence support following the first viral load test as diagnosed by ART physicians (3, 17).

**ART drug adherence:** We used the information from the card that was filled out by the physicians as good, fair, and poor adherence. This was assessed by patients' self-report about missed doses within a month, missing more than three doses from BID doses, and missing more than one dose from daily doses was considered poor adherence (18).

**Opportunistic infection:** People living with HIV and who developed either herpes zoster infection, candidiasis, cryptococcal meningitis, chronic diarrhea, encephalopathy, herpes simplex, Pneumocystis jiroveci pneumonia, pneumonia, pulmonary tuberculosis, extra-pulmonary tuberculosis, intestinal parasitosis, toxoplasmosis, or upper respiratory tract infections were considered as having opportunistic infections (19, 20).

### Data processing and analysis

First, data were checked for coding errors, completeness, and missing values. Then, data were entered into Epidata Manager version 4.0.2 and were analyzed using SPSS version 25. Descriptive statistics, such as frequencies, means, standard deviations (SD), and proportions were done for different variables. Bivariable logistic regression analyses were done to select a candidate variable for multi-variable logistic regression analysis. Variables with a *p*-value < 0.25 during the bivariable analysis were included in the multivariable logistic regression analysis. Model adequacy was done by the Hosmer and Lemeshow test (*p* = 0.62) and multicollinearity between independent variables was checked by variance inflation factor (VIF) (4). Variables with a *p*-value <0.05 were considered statistically significant determinants of virological failure in the final multivariable logistic regression model.

## Results

### Socio-demographic characteristics

In total, two hundred ninety-two patients (146 cases and 146 controls) were involved in this study. The mean age of cases was 37.14 (SD = 10.47) and 37.45 (SD = 8.372) years for control groups while ART was initiated. Furthermore, fifty-nine (40.4%) cases and 50 (34.2%) controls were in the age range of 35 years or less in their ART initiation time. Regarding marital status, eighty-eight (60.3%) cases and 95(65%) controls were married at ART initiation. Moreover, seventy-six (52%) cases and 54 (37%) controls were men. In total, fifty-five (37.7%) cases and 58 (39.7%) controls had not attended school (Table 1).

**Table 1:** Socio-demographic characteristics of patients positive with HIV on highly active antiretroviral therapy (HAART) at Mizan Tepi University Teaching Hospital (MTUTH), 2020.

**Variables**	**Categories**	**Cases**	**Controls**	***X^2^* test p-value**
		***N* (%)**	***N* (%)**	
Age at ART initiation	≤35 years	59 (40.4)	50 (34.2)	0.276
	>35 years	87 (59.6)	96 (65.8)	
Place of residence	Urban	94 (64.4)	94 (64.4)	0.489
	Rural	52 (35.6)	52 (35.6)	
Sex	Male	76 (52)	54 (37)	0.010
	Female	70 (48)	92 (63)	
Marital status	Never married	29 (19.8)	25 (17.1)	0.940
	Married	88 (60.3)	95 (65)	
	Divorced	13 (8.9)	11 (7.6)	
	Widowed	7 (4.8)	7 (4.8)	
	Separated	9 (6.2)	8 (5.5)	
Religion	Orthodox	75 (51.4)	76 (52.1)	0.489
	Muslim	27 (18.5)	20 (13.7)	
	Protestant	44 (30.1)	50 (34.2)	
Level of education	No education	55 (37.7)	58 (39.7)	0.352
	Primary	33 (22.6)	40 (27.4)	
	Secondary	38 (26)	37 (25.3)	
	Tertiary	20 (13.7)	11 (7.6)	

### Behavioral, medical, and nutritional related factors

Among the study participants, only 65 (44.5%) cases and 58 (39.7%) controls disclosed their HIV status. In this study, seventy-seven (52.7%) cases and 40 (27.4%) controls had a history of substance use, and 29 (19.9%) cases and eight (5.5%) controls had a history of poor adherence to ART drugs. Regarding the medical-related factors, 27 (18.5%) cases and 17 (11.6%) controls had CD4 count ≤200 cells/mm^3^ at the ART initiation, and 40 (27.4%) cases and 12 (8.2%) controls had an underlying chronic illness. Moreover, 95 (65.1%) cases and 52 (35.6%) controls had the baseline hemoglobin of <12 mg/dl. Furthermore, forty-three (29.4%) cases and 30 (20.5%) had a baseline BMI ≤18.4 kg/m^2^, while 96 (65.8%) cases and 101 (69.2%) controls had a baseline BMI of 18.5–24.99 kg/m^2^ (Table 2).

**Table 2:** The behavioral, clinical, and nutritional characteristics of patients with HIV treated at MTUTH in 2020.

**Variable**	**Categories**	**Cases**	**Controls**	***X*^2^ test p-value**
		**N (%)**	**N (%)**	
HIV status disclosure	Disclosure	65 (44.5)	58 (39.7)	0.108
	Not disclosed	81 (55.5)	88 (60.3)	
History of substance use	Yes	77 (52.7)	40 (27.4)	0.001
	No	69 (47.3)	106 (72.6)	
ART drug adherence	Poor	29 (19.9)	8 (5.5)	0.001
	Good	117 (80.1)	138 (94.5)	
CD4 count at ART initiation	≤200	27 (18.5)	17 (11.6)	0.106
	201=350	18 (12.3)	21 (14.4)	
	351-500	29 (19.9)	35 (24)	
	≥501	72 (49.3)	73 (50)	
Chronic illness	Yes	40 (27.4)	12 (8.2)	0.001
	No	106 (72.6)	134 (91.8)	
Baseline hgb (mg/dl)	<12	95 (65.1)	52 (35.6)	0.001
	≥12	51 (34.9)	94 (64.4)	
WHO staging at ART initiation	Stage 1	49 (33.6)	75 (51.4)	0.011
	Stage 2	55 (37.6)	41 (28)	
	Stage 3	35 (24)	28 (19.3)	
	Stage 4	7 (4.8)	2 (1.3)	
Functional status at ART initiation	Working	127 (87)	129 (88.4)	0.365
	Ambulatory	17 (11.6)	17 (11.6)	
	Bedridden	2 (1.4)	0 ()	
Baseline opportunistic infections	Yes	41 (28)	13 (8.9)	0.001
	No	105 (72)	133 (91.1)	
History of opportunistic infections during follow up	Yes	42 (28.8)	8 (5.5)	0.001
	No	104 (71.2)	138 (94.5)	
Baseline BMI (kg/m^2^)	≤18.4	43 (29.4)	30 (20.5)	0.058
	18.5–24.99	96 (65.8)	101 (69.2)	
	25–29.99	7 (4.8)	15 (10.3)	

### Drug-related factors

Among the study participants, 61 (41.8%) cases and 52 (35.6%) controls were <73 months on ART, and the majority of cases and controls had taken Current Procedural Terminology (CPT). Similarly, 27 (18.5%) cases and seven (4.8%) controls had a history of the restart after interruption. In total, sixty-four (43.8%) cases and 57 (39%) controls had taken the TDF-3TC-NVP regimen at ART initiation. In addition, 101 (69.2%) cases and 95 (65.1%) controls had experienced side effects (Table 3).

**Table 3:** Drug-related information of patients with HIV treated at MTUTH in 2020.

**Variable**	**Category**	**Cases**	**Controls**	***X*^2^ test p-value**
		***N* (%)**	***N* (%)**	
Duration on ART	<73 month	61 (41.8)	52 (35.6)	0.280
	≥73 month	85 (52.8)	94 (64.4)	
Cotrimoxazole preventive therapy	Yes	141 (96.6)	134 (91.8)	0.083
	No	5 (3.4)	12 (8.2)	
Restart after interruption	Yes	27 (18.5)	7 (4.8)	0.001
	No	119 (81.5)	139 (95.2)	
History of previous exposure to ART	Yes	3 (2)	0 (0.00)	0.082
	No	143 (98)	146 (100)	
ARV drug dispenses at initiation	1c = AZT-3TC-NVP	25 (17.1)	21 (14.4)	0.483
	1d = AZT-3TC-EFV	20 (13.8)	20 (13.8)	
	1e = TDF-3TC-NVP	64 (43.8)	57 (39)	
	1f = TDF-3TC-EFV	37 (25.3)	48 (32.8)	
History of side effects	Yes	101 (69.2)	95 (65.1)	0.455
	No	45 (30.8)	51 (34.9)	

### Determinants of virological failure

After adjusting for possible confounders, a multivariable logistic regression analysis showed that sex of the patient, history of substance use, baseline Hgb, restart after the interruption, poor drug adherence, and OI through follow-up had a statistically significant association with virological failure (Table 4).

**Table 4:** The bivariable and multivariable logistic regression analyses for factors associated with virological failure among first-line HAART at MTUTH in 2020.

**Variables**	**Categories**	**Cases**	**Controls**	**COR (95% CI)**	**AOR (95% CI)**	**p-value**
Sex	Male	76	54	1.85 (1.16, 2.95)	**1.89 (1.04, 3.47)**	0.038^*^
	Female	70	92	1	1	
History of substance use	Yes	77	40	2.96 (1.79, 4.75)	**2.67** (**1.40, 4.95**)	0.003^*^
	No	69	106	1	1	
Chronic illness^a^	Yes	40	12	4.21 (2.12, 8.51)	1.80 (0.61, 5.27)	0.28
	No	106	134	1	1	
Baseline Hgb	<12	95	52	3.36 (1.80, 5.35)	**3.22** (**1.82, 5.99)**	0.001^*^
	≥12	51	94	1	1	
WHO staging at ART initiation	Stage 1	49	75	1	1	
	Stage 2	55	41	2.05 (1.19, 3.53)	1.25 (0.63, 2.50)	0.52
	Stage 3	35	28	1.91 (1.03,3.53)	0.48 (1.91, 1.20)	0.11
	Stage 4	7	2	5.35 (1.07, 26.85)	0.44 (0.49, 4.08)	0.47
Opportunistic Infections through follow up	Yes	42	8	6.96 (3.07, 15.38)	**4.73** (**1.76, 12.11)**	0.001^*^
	No	104	138	1	1	
HIV status disclosure	Disclosed	65	58	1	1	
	Not disclosed	81	88	1.22 (0.67–2.35)	0.98 (0.46–1.89)	0.33
Restart after interruption	Yes	27	7	4.50 (1.89, 10.71)	**2.45** (**1.69, 7.35)**	0.001^*^
	No	119	139	1	1	
ART drug adherence	Poor	29	8	4.27 (1.87, 6.87)	**3.84** (**1.77, 5.95)**	**0.002**
	Good	117	138	1		
Baseline opportunistic infections	Yes	41	13	3.99 (1.74–5.33)	2.05 (0.91–4.78)	0.007
	No	105	133	1		

a Hypertension, heart failure, diabetes mellitus (DM), and chronic kidney disease (CKD).

b Bacterial pneumonia, tuberculosis (TB), Herpes zoster, and oral candidiasis, and the

* symbol indicate the variables significant in multivariable analysis at *p*-value < 0.05.

Bold values indicates statistically significant variables in the multivariable regression analysis.

In this study, virological failure was 1.89 times higher among men (adjusting odds ratio (AOR) = 1.89, 95% CI: 1.04, 3.47) compared with female patients. The odds of virological failure among those who had a history of substance use were 2.6 times higher compared with those who had no history of substance use (AOR = 2.67, 95% CI: 1.40, 4.95). Patients with Hgb <12 mg/dl were 3 times at higher odd (AOR = 3.22, 95% CI: 1.82, 5.99) compared with those who had baseline Hgb levels ≥12 mg/dl. In addition, patients with poor drug adherence (AOR = 3.84, 95% CI: 1.77, 5.95), and restart ART medication (AOR = 2.45, 95% CI: 1.69, 7.35) after interruption were at higher odds for virological failure. Moreover, the odds of virological failure were 4-fold higher (AOR = 4.73, 95% CI: 1.76, 12.11) among those who had a history of OI through the follow-up period compared with those who had no history of OI.

## Discussion

This study aimed to assess determinants of virological failure among patients on the first-line HAART at MTUTH. It showed that the sex of the patient, histories of substance use, baseline Hgb, restart after interruption of HAART, poor drug adherence, and opportunistic infections through follow-up were identified as determinants of virological failure.

The sex of the patient was identified as a determinant of virological failure. In this study, men were at a higher odd of developing virological failure. This finding is consistent with a study conducted in Spain (21) and the United States (22). However, this finding is inconsistent with a large cohort study conducted in Canada (23). The discrepancy could be explained by the differences in sample size and baseline clinical parameters. It could also be because men are more likely to use substances when compared with women (24); this, in turn, makes men more vulnerable to virological failure.

In this study, the odds of virological failure among those who had a history of substance use were higher compared with those who had no history of substance use. This finding is supported by the study conducted at Jimma (17), Addis Ababa (25), and Vietnam (26). This might be since using HAAT drugs and substances simultaneously would be prone to mental health problems resulting in poor adherence characterized by failure to take medication properly; moreover, interrupting the medication which leads finally to first-line ART treatment failure (27). Specifically, in our study area, this might be due to the more use of substances (such as, Bordea, Tella, Teji, Beer, and mostly younger groups used Khat as a recreational activity) since it is available easily in the area most as a cultural drink, and this might increase the negligence and carelessness of patients on using HAART properly.

Optimal treatment adherence is necessary for viral suppression and a good treatment outcome. This study identified poor drug adherence tripled the chance of virological failure among patients with first-line HAART. This finding is consistent with multiple previous shreds of evidence (3, 6, 28, 29). Those patients with poor drug adherence had no adequate drug concentration for sustained viral suppression. Similarly, our study showed patients with restarted after an interruption of HAART drugs had higher odds of developing virological failure. People often discontinue HAART drugs due to stigma, side effects, and lack of psychosocial support (30). Evidence showed interruption of HAART for a short period was associated with increased plasma viral load (pVL), which makes it difficult to achieve optimal viral suppression. This finding is in agreement with studies conducted in Ethiopia and the United Kingdom (30, 31).

Furthermore, the odds of virological failure among those who had baseline Hgb < 12 mg/dl were higher compared with those who had baseline Hgb ≥12mg/dl. This finding is supported by the study conducted at Jimma, Ethiopia (32), Cameron (33), and South Africa (34). This might be because patients with anemia are more likely to have advanced immune suppression and a higher rate of co-morbidities that could impose a negative effect on response to ARV treatments. Evidence from other studies also showed anemia at ART initiation is associated with increased mortality, disease progression (poor virological response), and reduced quality of life (32).

In addition, the odds of virological failure were higher among those who had a history of OI throughout the follow-up period compared with those who had no history of OI throughout the follow-up period. This finding is in line with the studies conducted in Jimma (17) and Arba Minch towns of Ethiopia (35) which showed patients with opportunistic infections, such as TB, appear to be at a higher risk of virological failure. This finding also consisted of a study conducted in Nigeria (36). Opportunistic infections could lead to depletion of CD4 cells and result in ART treatment failure (37). It could also be due to secondary infections impairing the immune system and providing a suitable environment for viral replication resulting in increased viral load or prone the patients to virological failure. Small sample size and missing of some important variables were some of the limitations of this study. Therefore, the study should be cautiously interpreted given the above limitations.

## Conclusion

The study revealed that the sex of the patient, history of substance use, baseline Hgb < 12 mg/dl, poor drug adherence, restart after the interruption, and having OI through the follow-up period were determinants of virological failure. Therefore, program implementation should consider gender disparity while men are more prone to virological failure. It is also imperative to implement targeted interventions to improve drug adherence and interruption problems in follow-up care. Moreover, patients with opportunistic infection and restart after HAART interruption need special care and attention.

## Data availability statement

The raw data supporting the conclusions of this article will be made available by the authors, without undue reservation.

## Ethics statement

The studies involving human participants were reviewed and approved by Mizan Tepi University College of Medicine and Health Sciences Ethics Committee. Written informed consent for participation was not required for this study in accordance with the national legislation and the institutional requirements.

## Author contributions

EY and AA conceived the study and supervised data collection. BB carried out the statistical analysis and drafted the manuscript. TY, AD, MA, ZA, AW, and GM participated in statistical analysis and reviewing of the manuscript. All authors contributed to the article and approved the submitted version.

## Conflict of interest

The authors declare that the research was conducted in the absence of any commercial or financial relationships that could be construed as a potential conflict of interest.

## Publisher's note

All claims expressed in this article are solely those of the authors and do not necessarily represent those of their affiliated organizations, or those of the publisher, the editors and the reviewers. Any product that may be evaluated in this article, or claim that may be made by its manufacturer, is not guaranteed or endorsed by the publisher.

## Abbreviations

AIDS: Acquired Deficiency Syndrome
ART: Antiretroviral Therapy
BMI: Body Mass Index
BSZ: Bench Sheko Zone
FMOH: Federal Ministry of Health
GDP: Global Domestic Product
HAART: Highly Active Antiretroviral Therapy
HIV-1: Human Immunodeficiency Virus 1
HIV: Human Immunodeficiency Virus
MTUTH: Mizan Tepi University Teaching Hospital
NNRTI: Non Nucleoside Reverse Transcriptase Inhibitor
OI: Opportunistic Infection
PICT: Provider imitative counseling and testing
PLWHA: People Living with HIV/AIDS
PMTCT: Prevention of Mother to Child Transmission
PVL: Plasma Viral Load
SNNPRS: Southern Nation Nationalities and People Regional State
TB: Tuberculosis
VCT: Voluntary counseling and Testing
WHO: World Health Organization.
